# From bedside to genetic analysis: New insights into pathophysiology of melanoma, basal cell carcinoma, and other cancers

**DOI:** 10.1111/srt.13832

**Published:** 2024-06-27

**Authors:** Alexander Zemtsov

**Affiliations:** ^1^ University Dermatology Center Muncie Indiana USA; ^2^ Department of Dermatology Indiana University School of Medicine Indianapolis Indiana USA

**Keywords:** abnormal splicing, cancer, environmental intron's damage and alterations, etiology, heterogenous nuclear ribonucleoproteins

## Abstract

**Objective:**

Patients with myotonic muscular dystrophy (MMD) were observed to have numerous basal cell carcinoma (BCC) and abnormal dysplastic nevi (DN) on non‐sun exposed skin. Simultaneously a large study published in the *Journal of American Medical Association* (JAMA) illustrated that patients with MMD have “overall” an increased risk for cancer development. Based on these findings, this author in 2010 postulated that dysregulation of RNA binding proteins (RBP), responsible for clinical manifestations of MMD, is also responsible for the development of BCC and melanoma.

**Methods:**

To report new research elucidating the etiology of melanoma, BCC, MMD‐induced cancers, and potentially other environmentally induced malignancies.

**Results:**

Dysregulation of RBP induces aberrant mRNA splicing; recent data indicates that abnormal mRNA splicing not just plays a key role in the pathogenesis of melanoma but is a hallmark of essentially all human malignancies.

**Conclusion:**

The author's hypothesis is that ultraviolet (UV) radiation induces DNA damage in intronic regions of a variety of genes. Furthermore, these UV‐induced abnormal DNA dimers, repeats and mutations interfere with normal mRNA splicing thus producing abnormal proteins. These abnormal proteins in turn activate oncogenic pathways such as hedgehog, MAP kinase, and WNT.

## 1 INTRODUCTION

Many decades ago Zemtsov observed a unique wheelchair‐bound young patient with Type 1 myotonic muscular dystrophy (MMD) with minimal solar elastosis who had numerous basal cell carcinoma (BCC) and dysplastic nevi (DN) in non‐sun exposed areas.^1^ The literature search revealed that the association between MMD and BCC has been previously reported by German dermatologists.^2^, ^3^ In subsequent years it became apparent that MMD is caused by CTG and CCTG DNA intron repeats located on DPMK (Type 1) and CNBP (Type 2) genes.^4^ Once transcribed these abnormal DNA intron repeats both sequester and interfere with normal function RNA binding proteins (RBP) (by producing hairpins and other abnormal slipped‐strand DNA configurations^5^) thus inducing abnormal mRNA splicing. The proteins produced by this abnormally spliced mRNA are responsible for clinical manifestations of Type 1 and 2 MMD. A pivotal study in the *Journal of American Medical Association* (JAMA) demonstrated that patients with MMD have an increased risk of developing cancer (overall and in selected anatomical areas).^6^

These observations led Zemtsov to postulate a hypothesis that ultraviolet (UV) radiation causes sequestration and/or malfunction RBP (aka and most commonly referred to now as heterogenous nuclear ribonucleoproteins [hnRNP]) leading to cutaneous carcinogenesis.^1^ Subsequently, large studies confirmed that MMD is associated with an increased risk of development of skin cancers.^7^, ^8^ As far as the etiology of cancer in MMD it is now believed to be a result of upregulation of the *Wnt/B‐*catenin pathway (Mueller and co‐authors)^6^, ^9^ or abnormally spliced mRNA products suppressing or inducing tumor‐suppressing and oncogenes (Zemtsov).^2^ Zemtsov being an Editor in Chief of this Journal and reviewing numerous manuscripts came across new research that made him modify his hypothesis and perhaps shed new light on many environmentally induced cancers including cutaneous carcinogenesis.

## 2 MATERIAL AND METHODS

In 2020, the authors published a review article on the role of the WNT signaling pathway in developing melanoma^10^ (Mueller mechanism). In March 2024, other investigators reported in their review article the pivotal role of abnormal splicing in the development and pathogenesis of melanoma^11^ (Zemtsov mechanism). The authors of this review article also emphasized that abnormal RNA splicing is a key factor in the pathophysiology of essentially all malignancies.^11^ This data collaborates with research done by Munoz and co‐authors that UV radiation produces an alternative DNA splicing.^12^ Munoz and co‐authors also briefly mention in their manuscript, published in 2009, that alternative splicing appears to play a role in cancer pathogenesis.^12^ This author decided to combine Mueller and Zemtsov mechanisms. Furthermore, reading carefully the article cited above,^11^ one mechanism for activation of BRAF oncogene in MAP kinase pathway is a point mutation in intron 8 (which leads to exons 4–8 not being translated).^11^ An extensive literature search revealed that UV radiation can induce a variety of point mutations.^13^ Obviously, DNA exposure causes the formation of a *variety* of abnormal DNA structures such as cyclobutane pyrimidine dimers (CPD), 6‐4 pyrimidine pyrimidone, and its Dewar isomers.^14^, ^15^, ^16^ Furthermore, there is a variety of nucleotide substitutions such as C to T, CC to TT, and G to T.^14^, ^15^, ^16^ More importantly at least some of these photoproducts can cause kinking in DNA structure producing *3D* hairpin and slipped strand structures—*the configurations analogous* to the ones formed by CTG DNA repeats in patients with MMD. I am fairly certain that no detailed NMR spectroscopic or electrophoretic analysis on these UV‐induced photoproducts was performed. However, these photoproducts *again* most likely are capable of forming hairpins and other abnormal DNA configurations similar to ones formed by MMD repeats.^5^

## 3 DISCUSSION

My *modified* hypothesis of UV‐induced cutaneous carcinogenesis is that UV radiation induces in a variety of introns (or even perhaps exons) abnormal DNA structures that have 3D similarity to the abnormal DNA configurations produced by MMD DNA repeats. These abnormal DNA structures interfere with RBP/hnRNPs causing abnormal mRNA splicing. The protein products of this abnormal splicing activate different oncogenic pathways. I *speculate* that perhaps the shape of DNA photoproduct in addition to its location (gene involved) determines what pathway gets activated; protein products of certain abnormal splicing activate the hedgehog pathway thus producing BCCs while others activate MAP kinase and WNT forming DN and melanomas. This hypothesis is supported by Munoz and co‐workers' data showing that UV‐induced alternative splicing occurs co‐transcriptionally but not post‐transcriptionally thus indicative of direct DNA genomic damage.^12^ Genome‐wide mapping of (preferably human or a mammalian) DNA UV damage by precise localization of cyclobutene pyrimidine dimers (CPD‐seq) may indicate genes involved in skin cancer pathogenesis.^17^ A similar technique (HS‐damage‐seq) analyzing the location of CPD and pyrimidine‐pyrimidone photoproducts in human cell cultures has also been developed.^18^

It is not unreasonable to suggest that similar mechanisms are present in other environmentally induced malignancies such as lung cancers. Clearly, additional experimental support and validation are required to confirm this hypothesis.

## Data Availability

The data that support the findings of this study are available on request from the corresponding author. The data are not publicly available due to privacy or ethical restrictions.
